# A randomised controlled trial of a program based on the theory of planned behavior to promote fruit and vegetable intake among schoolchildren: PROFRUVE study protocol

**DOI:** 10.1186/s12889-018-5748-3

**Published:** 2018-07-04

**Authors:** M. Arrizabalaga-López, D. Rada-Fernández de Jáuregui, M. P. Portillo, O. Martínez, I. Etaio, J. R. Mauleón, E. Echevarría, F. Gómez, V. M. Rodríguez

**Affiliations:** 1 Nutrition and Obesity Group, Dept. of Nutrition and Food Science, Faculty of Pharmacy, University of the Basque Country UPV/EHU, Paseo de la Universidad 7, 01006 Vitoria-Gasteiz, Spain; 2 Dept. of Preventive Medicine and Public Health, Faculty of Pharmacy, University of the Basque Country UPV/EHU, Vitoria-Gasteiz, Spain; 3 Dept. of Pharmacy and Food Science, Faculty of Pharmacy, University of the Basque Country UPV/EHU, Vitoria-Gasteiz, Spain; 4 Sensory Analysis Laboratory LASEHU, Dept. of Pharmacy and Food Science, Faculty of Pharmacy, University of the Basque Country UPV/EHU, Vitoria-Gasteiz, Spain; 5 Dept. of Sociology 2, Faculty of Pharmacy, University of the Basque Country UPV/EHU, Vitoria-Gasteiz, Spain; 6 Dept. of Physiology, Faculty of Pharmacy, University of the Basque Country UPV/EHU, Vitoria-Gasteiz, Spain; 7 Public Health Services, Dept. of Social Policies and Public Health, Vitoria-Gasteiz, Spain; 8 CIBERobn Physiopathology of Obesity and Nutrition, Institute of Health Carlos III, Vitoria-Gasteiz, Spain

**Keywords:** Intervention, Fruit, Vegetable, Intake, Behavioral theory, Children, Protocol

## Abstract

**Background:**

The PROFRUVE study is a controlled intervention based on the Theory of Planned Behavior (TPB), which follows those behavioral theories that have proved to be the most effective at changing infant fruit and vegetable (FV) intake pattern. The main purpose of the study is to evaluate the effectiveness of an intervention program in increasing FV consumption in schoolchildren aged 8 to 10 and based on TPB.

**Methods:**

Eligible classrooms within schools from Vitoria-Gasteiz (Basque Country, Spain) will be randomly assigned to the intervention (classrooms *n* = 4; children *n* = 86) or control (classrooms *n* = 4; children *n* = 86) group. The intervention group will receive 14 sessions of 60 min during an academic year (October to June). These sessions, designed by a multidisciplinary team, are based on TPB and are directed at modifying determinants of behavior (attitudes, subjective norms, perceived behavioral control and intention of consumption), and intake of FV itself. Both the process and the evolution of consumption and determinants of behavior will be evaluated (before, during, shortly after and a year after) using validated surveys, 7 day food records, 24 h reminders and questionnaires.

**Discussion:**

This study will provide a valid and useful tool to achieve changes in the consumption of FV at school level. A negative result will be helpful in redefining new strategies in the framework of changing habits in the consumption of FV.

**Trial Registration:**

This study has been retrospectively registered at ClinicalTrials.gov. Identifier: NCT03400891. Data registered: 17/01/2018.

## Background

Developed societies are characterized by their unhealthy eating patterns, which contribute to the high prevalence of overweight and obesity according to WHO. As a consequence it is a massive costly public health matter. The ALADINO 2015 study has shown overweight rates of 23.4% and obesity rates of 18.1% among 6–9 years old Spanish children. The data from the same study relating to the Basque Country indicate a high prevalence: 24.1% overweight among girls and 21.8% among boys, and 8.3% obesity among girls and 14.1% among boys [1]. Our research group analyzed the youth population of Vitoria-Gasteiz, the capital of the Basque Country, located in the north of Spain. These data showed that 22% of the schoolchildren aged 6 to 17 were overweight and 3.2% obese (unpublished data of City Council Nutritional Observatory, 2007).

Bad eating habits are at the head of the most frequent causes of weight problems [2]. Sufficient intake of fruit and vegetable (FV) has direct repercussions on dietary energy intake. It is a key in eating habit which promotes healthy weight because of its low caloric value and its fiber supply in the diet [3, 4]. In addition, increased FV intake has been shown to have a beneficial effect on bone density [5], cardiovascular health [6–8], diabetes [9], cancer [10] and metabolic syndrome [11].

According to the ENALIA Study, among Spanish youngsters under 17 years, only 31.7% use to eat FV daily [12]. The latest study carried out in the Basque Country, showed that children and youngsters aged 4 to 18 usually eat 98.9–114.2 g of fruit per day (boys and girls respectively) and 90.3–86.5 g of vegetables per day (boys and girls) [13]. This is a long way from the recommended 400 g of FV per day [14]. In Vitoria-Gasteiz, only two in ten children and adolescents (aged 6 to 17) consume the recommended three servings of fruit and only one in ten the recommended two servings of vegetables daily. Average intake of fruit was 1.8 servings/day and 0.8 servings/day for vegetables (unpublished data of City Council Nutritional Observatory, 2007). For this reason, promotion of sufficient intake of FV must be a priority objective in the promotion of healthy eating habits.

Taking into account that the habits acquired in childhood tend to remain in adulthood [15] and that food education and healthy lifestyle adequately raised are effective in the infant population, nutrition education seems to be the ideal tool for increasing FV intake [16]. For this purpose the school environment is the perfect scenario for training both healthy eating and lifestyle healthy habits [17, 18]. It is of paramount importance to involve all those engaged in the education of children: parents and teachers [19, 20] and even classmates [21].

However, the food environment has become increasingly complex and just to transfer information on eating and lifestyle habits is not enough to modify them [22, 23]. Other intervention approach is to use behavioral theories to understand and modify food choice behavior [23–25]. One of the most widely used theories backing these types of interventions is the Theory of Planned Behavior (TPB). According to this theory eating behavior and intention of consumption determinants are both personal and environmental. Specifically, the TPB states that attitude, subjective norms and perceived control are determinants that influence both the intention to consume and the action of consuming food [24].

According to the scientific bibliography, the most effective programs that have attempted to modify the pattern of infant feeding in terms of FV have been those that contemplate the determinants of eating behavior [25–27]. In fact, with regard to a meta-analysis that studied the influence of behavioral theory-based interventions on children’s FV intake, the effectiveness of theory-based and non–theory based studies differed significantly. Moreover, the intervention was more effective when it was theory-based than non–theory based, irrespective of the number of theories used for the interventions. Furthermore it was concluded that the quality of the study is more important than the theory itself or the number of theories used [27].

### Hypothesis

An intervention program to increase FV intake in schoolchildren aged 8 to 10 and based on TPB will produce changes in attitude, subjective norms, perceived behavioral control and intention of eating FV, which in turn will modify behavior, thus increasing FV intake.

### Objectives

The main objective of this study is to evaluate the effectiveness of an intervention program based on TPB in increasing FV intake in schoolchildren aged 8 to 10.

Secondary objectives of the study are (i) to evaluate the change in FV intake in the study population after intervention, (ii) to examine the impact of the intervention program on behavioral determinants, (iii) to analyze the association of the behavioral determinants with FV intake, and (iv) to study the relationship between social demographic variables and the effectiveness of the program.

## Methods

### Design

A cluster randomized controlled trial will be carried out over an academic year at school level. Classrooms from different schools and schools themselves will be the clusters of the study. Clustering was chosen because this is the easiest way to implement an intervention program at school level. In this way the phenomenon of contamination between children from the same classrooms is avoided.

Whole classes (a minimum of 26 children per classroom) from different schools will be part of the intervention or control group. The control group allows us to analyze the direct effect of the intervention and to discard an increase of FV intake by seasonality (first measurements will be in October and final ones in June) or other factors. The study was approved by the Ethic Committee of the University of the Basque Country (CEISH/262/2014/RODRIGUEZRIVERA) and all parents or legal guardians and school directors and teachers will be sent an informed consent before the study starts. Written informed consents will be sent to parents or legal guardians through the children. After having received consents, the collection of the first data will be carried out followed by the randomized allocation of classrooms.

### Participants

The study will be conducted in schools from the capital of the Basque Country: Vitoria-Gasteiz. The classrooms within schools for this study will be randomly selected from a list given by the City Council of Vitoria-Gasteiz. Contact between the research group and schools will be made by the City Council.

The schools will be chosen to be representative in terms of ethnicity and social economical status. The inclusion criteria for this sample are: all schools from Vitoria-Gasteiz containing enough children aged 8 to 10 per classroom (a minimum of 26 children) and orchard. The exclusion criteria are: special schools and schools that are carrying out some other program related to healthy eating habits promotion in the same period. Having carried out previous nutritional programs will not be considered an exclusion criterion because the majority of schools from Vitoria-Gasteiz have participated in different programs organized by the City Council.

### Sample size

In order to provide a power of 90% to detect an effect size (Cohen’s d) of at least 0.5 servings/day, 172 participants (86 in the intervention group and 86 in the control group) are required. Based in the dropout of similar studies [28–30], the final sample size is increased by 20% to reach 206 children (8 classrooms with a minimum of 26 children per class).

### Randomization and blinding

After baseline data collection, eligible classrooms from different schools will be randomly assigned to the intervention or the control group, by a random sequence generated using IBM-SPSS Statistical software. The personnel responsible for randomizing will be blinded to participants. Schools (directors, teachers and children) and families also will be blinded to their intervention or control group.

### Study procedure

**Fig. 1:**
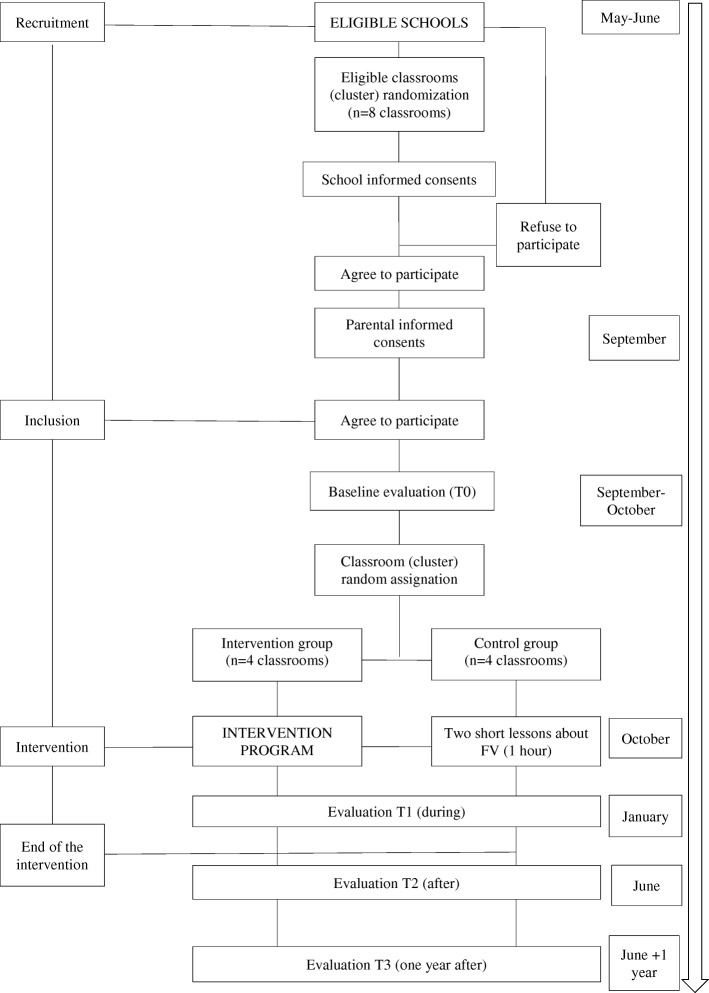
Diagram of the study timeline

The intervention program will last one academic year. After randomization, meetings will be held with teachers of each classroom to describe the specific program to be carried out by each classroom without telling them which group they belong to (control or intervention) and to set the dates of sessions and evaluation procedures. Teachers of the intervention groups will receive some training, as they are models for children. All intervention and control sessions will be held by the same nutritionist, and data collection at baseline (T0: September), during (T1: January) and after (T2: June) intervention will be performed by the same researcher. To check any long-term effect, the measurements will be carried out one year after the end of the intervention (T3: June). Diagram of the study timeline is presented in Fig. 1

.

### Intervention design

Following baseline data collection and randomization, the intervention group will start receiving program lessons every 15 days. Intervention group will receive 14 sessions of one hour over an academic year, from a nutritionist.

A multidisciplinary team designed the program. All materials, sessions and the program design are based on TPB and are respectful of gender, culture or religion. This theory states that people’s behavior is determined by their intentions, which in turn are influenced by attitudes, subjective norms and perceived behavioral control [24].

- Behavior: the action of eating FV.
- Intention: perceived likelihood of eating FV.
- Attitude: favorable or unfavorable judgments about eating FV. These judgments are determined by people’s beliefs, experiences and knowledge.
- Subjective norms: beliefs that most people who are important to children either approve or disapprove of them eating FV.
- Perceived behavioral control: perceptions of how much control they have over the behavior (action of eating FV).

In this study, these different variables are worked as described in Table 1.

**Table 1:** Techniques to work behavioral determinants

Determinants of TPB	Behavior change technique	Example of how it is applied in the program
Attitude	Provide information on the behavior-health relationship	More FV ... “Why?” “Because they are healthy” They contribute ... “Fiber helps your gut work!”
	Work on positive / negative consequences of behavior (action / inaction)	The consequences of behavior are worked on according to the health benefits of various components of VF “To Do or Not To Do”
	Provide information about the relationship between pleasure and behavior	More information given about we eat what we like More FV ... “Which?” “The ones you like!”
	Work the ability to get pleasure through eating FV	The capacity of discriminating sensorial analysis is worked on, encouraging exploration “Explore with your senses” “Enjoy the ones you like!”
Subjective norms	Provide information about others behavior	Record the consumption of FV of all classmates on a sheet that all can see (“Fruitmeter”)
Perceived behavioral control	Work on instructions to increase autonomy or ability	Instructions are provided to make it easy reaching goals such as 5 a day: “If you eat fruit for breakfast... you’re already in way 1 out of 3!” “Food and dinners are times ... with vegetables on your plates!”
	Work on situations that promote autonomy	Situations related to autonomy that promote the consumption of FV are practiced: participating in processes involved in producing, selling and preparing FV, e.g. cultivating, choosing and buying, cooking). “Get to know the orchard” “Participate! Choose and buy” “Have fun cooking”

Combining different theories in the program was discarded. This choice is based in a meta-analysis that analyzed the influence of behavioral theories on FV intervention effectiveness among children that found no association between the number of theories and consumption [27].

To make the program more effective, sessions were designed based on learning taxonomies [31], active learning methodologies and persuasion techniques (ELM: Elaboration Likelihood Model) [32]. All sessions have their own script for the nutritionist involved and all the scripts have the same structure (objectives, methodology, argument and resources). The script will be followed by the nutritionist with the aid of an audiovisual presentation, sheets for students, sheets for families, a goal-diary, FV’s folder, album and stickers, and material for specific sessions (e.g. FV as rewards at the end of each session and the “Fruitmeter”).

The proposed strategy has three axes of action: a) school activities, b) outside activities and c) home activities.

- School activities: Nine sessions will be held within the school (7 within the classroom and 2 FV cooking sessions in the dining room). Every month a “Fruitmeter” session will take place. In this activity, each classroom should write down their group FV mean intake using a tool called the “Fruitmeter”, which works by social pressure (subjective norms) to reach the objective of eating more FV. The “Fruitmeter” has poster format and will be hung in each classroom.
- Outside activities: Two visits to local product markets and two visits to local farmers. The last session will be held in a local theatre as the final FV program party.
- Home activities: a goal-diary which works with objectives related to FV consumption, e.g. to try a new vegetable this week or to eat fruit for breakfast three times per week, will be filled in at home with participants’ families. All worksheets that children use at school will be noted when they are reviewed at home with families.

The control group only will receive two one-hour lessons during the academic year with general information about the benefits of FV intake. These sessions will be given by a nutritionist and will not be based on behavioral theories.

### Outcomes

#### Primary outcome: Quantitative outcome

- FV intake: FV (excluding potatoes and legumes) consumption will be measured by a validated self-fulfilling food record [33]. It provides information about standard servings of FV intake for 7 days, and it must be filled out by parents or legal guardians. To control self-completed food record data reliability, a researcher will make telephonic 24-h dietary recalls (goal-standard) on one randomly chosen day during the week in which the record is being filled.

#### Secondary outcomes: Qualitative outcomes

- Determinants of eating behavior: evolution of determinants proposed (attitude, subjective norms and perceived behavioral control) will be analyzed by a questionnaire designed according to bibliographic proposals [34]. Each determinant will be measured by a minimum of 3 items that will be answered by children marking a number on a 1–5 scale from “totally disagree” to “totally agree”. Validity of the survey will be checked by its internal consistency (Cronbach’s alpha).

- Social demographic outcomes and families’ FV consumption habits: data will be collected by a questionnaire designed by the Department of Sociology 2 of the University of the Basque Country to obtain the following information:

- Parental educational attainment
- Parental employment situation
- Their perceived importance of their children’s FV intake
- Their level of knowledge concerning FV recommended intake
- FV availability and accessibility at home
- Where children have their main meal
- Children’s TV watching habits during meals
- Parents’ FV consumption habits.

Codified surveys will be sent by means of the children inside codified sealed envelopes. These will be filled out at home by parents and returned to school. Questionnaires of behavior determinants of behavior will be completed in 20 min by children at school under the supervision of a nutritionist. All the questionnaires will be completed at the three times described previously (T0, T1, T2) during the academic year and one year after intervention will be finished (T3).

### Statistical analysis

Descriptive statistics will be carried out to describe baseline characteristics of intervention and control group to assure comparability between groups.

Intra-group and inter-group FV intake differences at T0, T1, T2 and T3, will be analyzed by Student’s *t* test or non-parametric tests (Wilcoxon signed-rank test and Mann–Whitney U test). The same tests will be carried out to study the evolution of determinants of eating behavior (attitude, subjective norms, perceived behavioral control and intention) at different times.

To understand the relationship between determinants of behavior and behavior itself (FV consumption), multiple linear regression and structural equation modeling (SEM) analysis will be done at different times. Covariance of classic relationship of TPB determinants will be proposed. It will allow us to check the validity of the proposed model, as well as the influence of each determinant on the others and its significance. Changing models will be made by using difference on TPB determinants between different times.

Model validity will be established by comparative fit index (CFI), root mean square-error of approximation (RMSEA) and chi square/degrees of freedom (χ2/df). A good model will be determined by high values of CFI (> 0.90), low values of RMSEA (< 0.10) and χ2/df values from 1 to 3 [35, 36].

Multiple linear regression will be used to study the effect of social demographic variables in the effectiveness of the intervention.

Statistical analyses will be made using STATA 14.0, IBM-SPSS 24.0 and AMOS 24.0 statistical software. IC of 95% and significance level of *p* < 0.05 will be assumed.

## Discussion

An increase in the consumption of FV at the end of the program may indicate that the proposed program is a valid and useful tool for achieving changes in their consumption. A negative result will be helpful in redefining new strategies in the framework of changing habits in the consumption of FV. In addition, the one-year analysis (T3) would indicate the adherence to the program through time.

Thanks to SEM analysis, we will be able to measure the influence of different determinants on the process of change in the habit of eating FV, an important and new advance for future designs of more focused programs.

### Trial status

This study is ongoing until December 2018.

## Abbreviations

FV: Fruit and vegetable
SEM: Structural equation modeling
TPB: Theory of planned behavior
